# Decreased Diversity of the Oral Microbiota of Patients with Hepatitis B Virus-Induced Chronic Liver Disease: A Pilot Project

**DOI:** 10.1038/srep17098

**Published:** 2015-11-26

**Authors:** Zongxin Ling, Xia Liu, Yiwen Cheng, Xiawei Jiang, Haiyin Jiang, Yuezhu Wang, Lanjuan Li

**Affiliations:** 1 Collaborative Innovation Center for Diagnosis and Treatment of Infectious Diseases, State Key Laboratory for Diagnosis and Treatment of Infectious Diseases, the First Affiliated Hospital, School of Medicine, Zhejiang University, Hangzhou, Zhejiang, 310003, China; 2 Intensive Care Unit, the First Affiliated Hospital, School of Medicine, Zhejiang University, Hangzhou, Zhejiang, 310003, China; 3 Shanghai-MOST Key Laboratory of Health and Disease Genomics, Chinese National Human Genome Center at Shanghai, Shanghai 201203, China

## Abstract

Increasing evidence suggests that altered gut microbiota is implicated in the pathogenesis of hepatitis B virus-induced chronic liver disease (HBV-CLD). However, the structure and composition of the oral microbiota of patients with HBV-CLD remains unclear. High-throughput pyrosequencing showed that decreased oral bacterial diversity was found in patients with HBV-CLD. The Firmicutes/Bacteroidetes ratio was increased significantly, which indicated that dysbiosis of the oral microbiota participated in the process of HBV-CLD development. However, the changing patterns of the oral microbiota in patients with HBV-induced liver cirrhosis (LC) were almost similar to patients with chronic hepatitis B (CHB). HBV infection resulted in an increase in potential H_2_S- and CH_3_SH-producing phylotypes such as *Fusobacterium*, *Filifactor*, *Eubacterium*, *Parvimonas* and *Treponema*, which might contribute to the increased oral malodor. These key oral-derived phylotypes might invade into the gut as opportunistic pathogens and contribute to altering the composition of the gut microbiota. This study provided important clues that dysbiosis of the oral microbiota might be involved in the development of HBV-CLD. Greater understanding of the relationships between the dysbiosis of oral microbiota and the development of HBV-CLD might facilitate the development of non-invasive differential diagnostic procedures and targeted treatments of HBV-CLD patients harbouring specific oral phylotypes.

A chronic hepatitis B virus (HBV) infection, a major global public health problem, may evolve to cirrhosis and hepatocellular carcinoma (HCC), which affects an estimated 350 million people worldwide and results in more than 0.5–1 million deaths per year. Increasing evidence has shown that an altered profile of the human gut microbiome is associated with cirrhosis and its complications, such as hepatic encephalopathy, spontaneous bacterial peritonitis, and other infections^1^,^2^,^3^. The intestinal microbial community may act as an independent organ in regulating the metabolic balance of an entire body, which may affect the prognosis of hepatitis B virus-related liver cirrhotic patients^4^. In addition to affecting bacterial translocation to the gut and intestinal inflammation, the microbial metabolites produced in a dysbiotic intestinal environment play a vital role in the pathogenesis of liver disease^5^. Chou *et al*. showed that establishing the gut microbiota that is present in adult mice stimulated liver immunity, resulting in rapid HBV clearance^6^. As is the case in the gut, the oral cavity is also home to microbial communities, which has important implications for human health and disease. More than 700 species or phylotypes are found in the oral cavity, of which approximately 35% have not been cultured^7^. The interaction between the members of the oral microbiota and the host is associated with the level of oral health^8^,^9^,^10^,^11^ and also contributes to the development of several systemic chronic diseases, such as inflammatory bowel disease^12^, pancreatic diseases^13^, obesity^14^, and atherosclerosis^15^. However, the oral microbiota of patients with HBV-induced chronic liver disease (HBV-CLD) has not been previously investigated. The results of our previous metagenomic study suggested that commensal oral bacteria can invade the gut of patients with liver cirrhosis, which contributed to the small-intestinal bacterial overgrowth associated with liver cirrhosis^2^. Sheehy *et al*. demonstrated that the oral microbiota of children undergoing liver transplantation changed significantly immediately post-transplantation but was restored to baseline levels at 3 months post-transplantation^16^. However, the oral microbiota of healthy controls and patients with HBV-CLD has not yet been directly compared. Because the progression from chronic hepatitis B (CHB) to liver cirrhosis is a chronic and lengthy process, the aim of the present study was to characterize the differences in the composition of the oral microbiota of HBV-CLD patients compared with those of healthy controls using massively parallel barcoded 454 pyrosequencing, which would be helpful in understanding the relationships between the oral microbiota and HBV-CLD at a deeper level and in facilitating the development of novel non-invasive differential diagnosis procedures and targeted treatments of HBV-CLD patients harbouring specific oral bacteria.

## Results

### Decreased bacterial diversity of the oral microbiota associated with HBV-CLD

**Figure 1:**
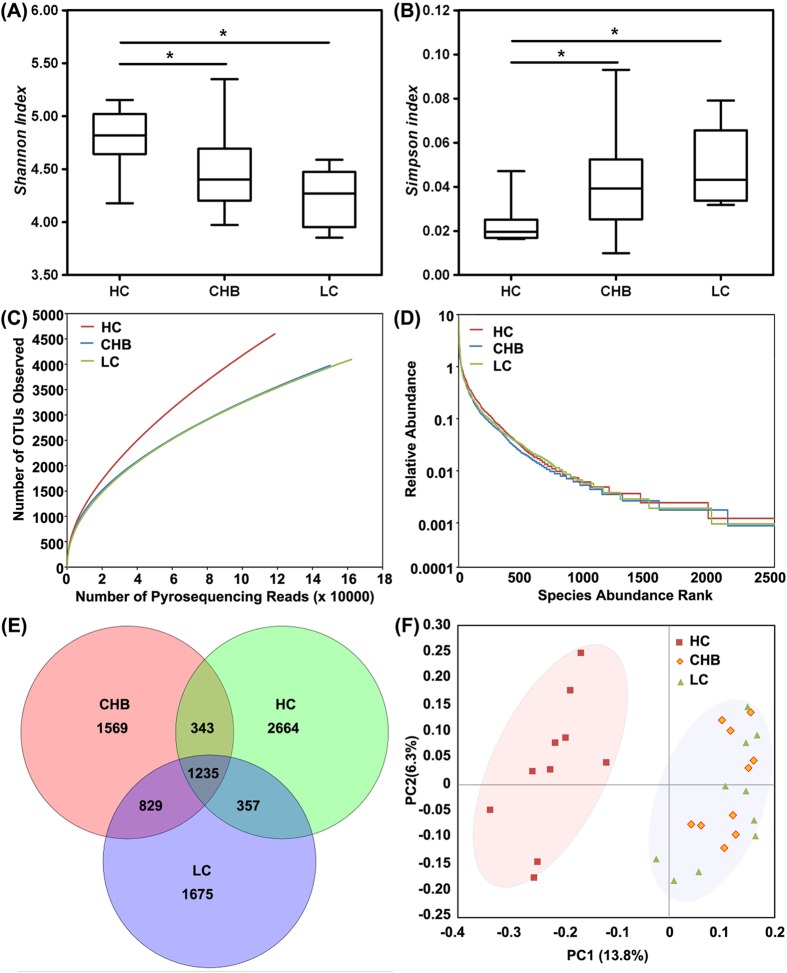
Comparison of the structures of the oral microbiota of the healthy control, CHB and LC groups. Shannon (**A**) and Simpson (**B**) indices were used to estimate the level of diversity (i.e., a combined assessment of the number of 97% similar bacterial taxa and their abundance) of the oral microbiota of children (data shown as the mean values and the SEM). Rarefaction curves were used to estimate the richness (at a 97% similarity level) of the oral microbiota of the three groups (**C**). The vertical axis shows the number of OTUs that expected to be found after sampling the number of tags or sequences shown on the horizontal axis. Rank abundance curve of the bacterial OTUs derived from the three groups (**D**). Venn diagram illustrating the overlap of the OTUs identified in the oral microbiota of the three groups (**E**). A principal-coordinate analysis plot of the oral microbiota based on the results of the unweighted UniFrac metric (**F**).

In our present pyrosequencing study, 612,922 raw sequences with a median read length of 467 base pairs (ranging from 201 to 540) were obtained. After quality trimming and chimera checking was conducted, 430,753 high-quality reads remained, accounting for 70.3% of the valid reads with an average of 14,358 reads (ranging from 7,266 to 20,529) per barcoded sample recovered for downstream analysis. The summary information is shown in Table 1, and the detailed characteristics of each sample are shown in Table S1. In total, 8,672 unique sequences were obtained from the three groups, representing all of the phylotypes present in the oral microbiota. The Good’s coverage values were high for all of the sequences in the three groups, indicating that the sequencing depth was sufficient for the investigation of the oral microbiota of HBV-CLD patients. Indicators of the alpha diversity, such as the Shannon and the Simpson indices, demonstrated that the level of diversity of the oral microbiota of the HBV-CLD patients was significantly lower than that of the healthy controls (p < 0.01, Fig. 1A,B). However, the diversity indices of the CHB and LC patients were not significantly different (p > 0.05). Rarefaction analysis estimates showed that the species richness of the oral microbiota of HBV-CLD patients tended to be lower than that of the healthy controls, whereas those of the CHB and LC patients were highly similar (Fig. 1C and Figure S1). Based on the results of the operational taxonomic units (OTUs) analysis, the rank-abundance curves for the bacterial communities of the healthy control and HBV-CLD groups exhibited similar patterns (Fig. 1D). A few species were abundant and the rare species accounted for the long right-hand tail of the rank-abundance curve. In addition, a Venn diagram displaying the overlapping OTU data for the three groups was developed to better understand their shared richness. This analysis showed that only 1,235 of the 8,672 OTUs accounting for the total richness were common to all of the samples, whereas 2,064 of 6,008 OTUs were common to the samples obtained from the CHB and LC patients (Fig. 1E). These data demonstrated that approximately 1,000 of the OTUs identified in the healthy-control samples were not detected in HBV-CLD-patient samples. To evaluate the extent of similarity of the microbial communities, the beta-diversity values were calculated using the unweighted UniFrac method and principal coordinate analysis (PCoA) was performed. Despite significant inter-individual variation, the oral microbiota of the HBV-CLD patients and the healthy controls could be clearly separated using PCoA (Fig. 1F), whereas the oral microbiota of the CHB patients could not be differentiated from that of the LC patients. These results indicated that a decreased level of bacterial diversity was found in the oral microbiota of the HBV-CLD patients compared with that of the healthy controls, whereas the overall structures of the oral microbiota of the CHB and LC patients were not significantly different.

### Alterations in the composition of the oral microbiota associated with HBV-CLD

**Figure 2:**
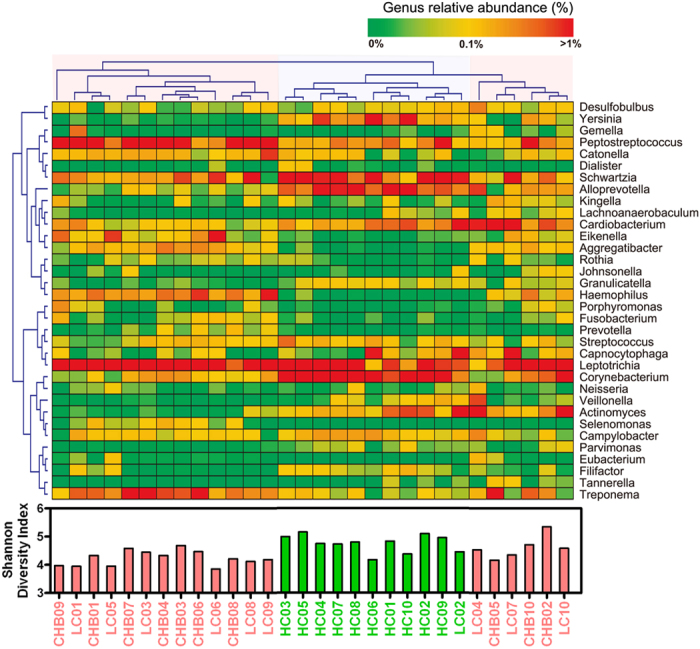
Heatmap indicating the genus-level changes in the healthy control, CHB and LC groups. The legends below the heatmap represent each participant. The relative abundance of the bacteria in each genus is indicated by a gradient of colour from green (low abundance) to red (high abundance). Complete linkage clustering of the samples was based on the genus-level composition and abundance of the oral microbiota. Three distinctive clusters were found in the oral microbiota, which was significantly associated with the Shannon index value.

The taxonomy of the oral microbiota was assessed via a taxon-dependent analysis using the RDP classifier. The vast majority of the sequences were assigned to six bacterial phyla (Actinobacteria, Bacteroidetes, Firmicutes, Fusobacteria, Proteobacteria and Spirochaetes), whereas sequences of representatives of Acidobacteria, Candidatus Saccharibacteria, Chloroflexi, Deinococcus-Thermus, Elusimicrobia, Gemmatimonadetes, Latescibacteria, Parcubacteria, Planctomycetes, Synergistetes, Tenericutes and one candidate division (SR1) were also identified but occurred in much lower proportions. Significant differences were observed in the relative proportions of members of the four major phyla (Actinobacteria, Bacteroidetes, Firmicutes and Spirochaetes) and the four non-dominant phyla (Candidatus Saccharibacteria, Chloroflexi, Tenericutes and SR1) in the healthy-control and HBV-CLD samples. The Firmicutes/Bacteroidetes ratios of the healthy control and HBV-CLD samples were significantly different (0.5143 ± 0.1980 vs. 1.4293 ± 0.8897, p < 0.05). The sequences obtained from the oral microbiota samples could be classified into 248 genera, with members of 104 genera found in the healthy-control samples, members of 167 genera found in the CHB-patient samples and members of 211 genera found in the LC-patient samples. Consistent with our rank abundance curves and based on the data obtained using a greater sequencing depth, members of the majority of the genera were present in low abundance in the oral microbiota samples. Of the total number of genera represented in the oral microbiota, members of 14 abundantly represented genera (>1% of the total DNA sequences) were detected in the healthy-control samples, whereas members of 16 and 18 abundantly represented genera were detected in the CHB- and LC-patient samples, respectively. The heatmap showed the correlations between the participants and the abundance levels of selected genera that were represented in the microbiota samples (Fig. 2). Consistent with the values for the alpha-diversity indices such as the Shannon index and those for the beta-diversity metrics such as PCoA, clustering analysis of the represented genera highlighted apparent differences in their distributions according to the health status of the host. It was obvious that an aberrant composition of the oral microbiota was associated with HBV-CLD.

To identify the specific bacterial taxa associated with HBV-CLD, we compared the composition of the oral microbiota of healthy controls and HBV-CLD patients using the linear discriminant analysis effect size (LEfSe) method. A cladogram representative of the structure of the oral microbiota and the predominant bacteria in the healthy-control and HBV-CLD-patient samples is shown in Figure S2 A; in which the largest differences in the taxa represented in the two communities are displayed. The changes in the composition of the oral microbiota after HBV infection were also explored using the Mann-Whitney U test at different taxon levels. The representation of 29 bacterial taxa at the family level and 42 bacterial taxa at the genus level was changed significantly by HBV infection (p < 0.05). Members of bacterial taxa belonging to the Bacteroidetes, Actinobacteria and Proteobacteria were enriched in the healthy-control samples, whereas those belonging to the Firmicutes and Spirochaetes were enriched in the HBV-CLD-patient samples which could be used as biomarkers for discriminating patients with an HBV infection (Figure S2 B).

**Figure 3:**
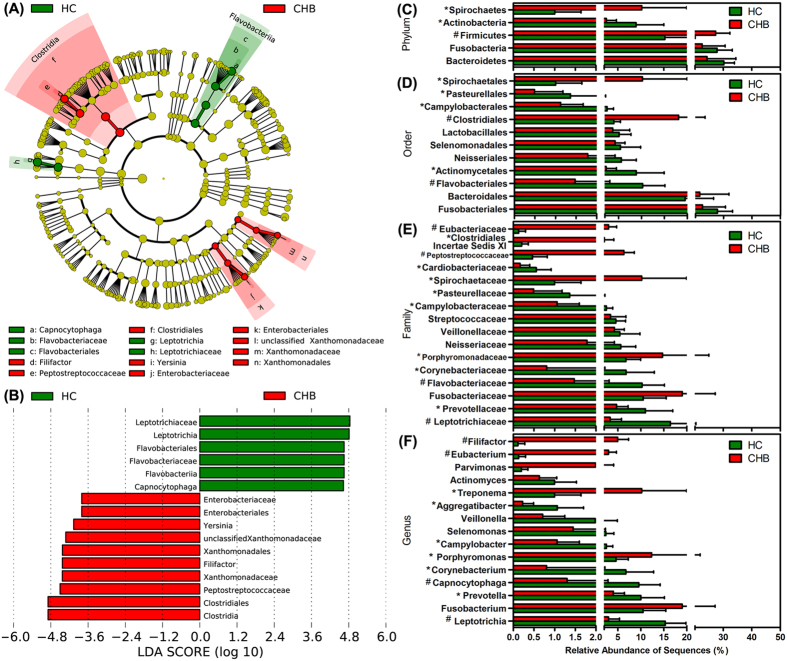
LEfSe was used to identify the most differentially abundant taxa in the healthy control and CHB patient samples. Taxonomic cladogram obtained using LEfSe analysis of the 16S sequences. (Red) CHB patient-enriched taxa; (Green) healthy-control enriched taxa. The brightness of each dot is proportional to its effect size (**A**). The healthy control-enriched taxa are indicated with a positive LDA score (green), and the CHB patient-taxa have a negative score (red). Only the taxa meeting a significant LDA threshold value of >2 are shown (**B**). Comparison of the relative abundance at the bacterial phylum (**C**), order (**D**), family (**E**) and genus (**F**) levels in the HC and CHB groups; * indicates p < 0.05; ^#^ indicates p < 0.01.

The differences in the overall composition of the oral microbiota of the healthy controls and CHB patients were also investigated using the LEfSe method (Fig. 3A,B). At the phylum level, the proportion of Firmicutes and Spirochaetes members was increased in the CHB-patient samples relative to that of the healthy controls, whereas the proportion of Actinobacteria members was decreased (Fig. 3C). At the order level, the proportions of the Clostridiales and Spirochaetales members were increased in the CHB-patient samples, whereas the proportion of the Flavobacteriales, Actinomycetales, Campylobacterales and Pasteurellales members were decreased (Fig. 3D). At the family level, members of the *Porphyromonadaceae*, *Spirochaetaceae*, *Peptostreptococcaceae*, *Clostridiales Incertae Sedis* XI and *Eubacteriaceae* were prevalent in the CHB-patient samples, whereas members of the *Leptotrichiaceae*, *Prevotellaceae*, *Flavobacteriaceae*, *Corynebacteriaceae*, *Campylobacteraceae* and *Pasteurellaceae* were enriched in the healthy-control samples (Fig. 3E). At the genus level, the representation of 10 genera was dramatically different in the CHB-patient samples compared with that of the healthy-control samples, with that of *Leptotrichia*, *Prevotella*, *Capnocytophaga*, *Corynebacterium*, *Campylobacter* and *Aggregatibacter* decreased and that of *Porphyromonas*, *Treponema*, *Eubacterium* and *Filifactor* increased (Fig. 3F). In addition, the overall composition of the oral microbiota of the LC patients was also significantly different from that of the healthy controls (Figure S3 A,B). At the phylum level, the proportion of members of Firmicutes was increased and that of Actinobacteria was decreased in the LC-patient samples compared with those of the healthy-control samples, similar to the altered pattern in the CHB-patient samples (Figure S3 C). At the order level, the proportions of members of Clostridiales, Enterobacteriales, Pseudomonadales and Rhizobiales were significantly increased compared with those of the healthy-control samples, whereas those of Flavobacteriales, Actinomycetales and Cardiobacteriales were decreased (Figure S3 D). With the exception of the changes observed in CHB patients, members of the *Fusobacteriaceae*, *Actinomycetaceae*, *Moraxellaceae*, *Xanthomonadaceae, Staphylococcaceae*, *Pseudomonadaceae* and *Rhizobiaceae* were more prevalent in the LC-patient samples than in the healthy-control samples (Figure S3 E). At the genus level, the proportions of members of the *Fusobacterium*, *Catonella*, *Eubacterium*, *Filifactor*, *Yersinia* and other 11 non-dominant genera were significantly increased in the LC-patient samples compared with those of the healthy-control samples and those of the *Leptotrichia*, *Prevotella*, *Capnocytophaga*, *Selenomonas*, *Aggregatibacter*, *Lachnoanaerobaculum*, *Cardiobacterium* and *SR1 genera incertae sedis* were dramatically decreased (Figure S3 F). Collectively, these differences in the compositions of the oral microbiota revealed the dysbiosis involved in the development of HBV-CLD.

### Discriminating the oral microbiota of CHB and LC patients

**Figure 4:**
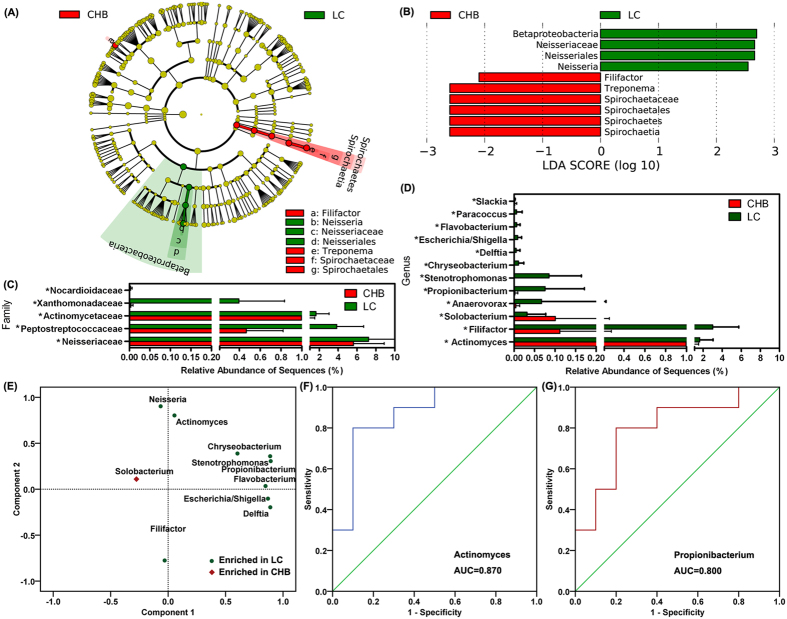
Taxonomic differences in the oral microbiota of the CHB and LC groups. Cladogram representing the features of the bacterial hierarchies determined using the LDA-based model (**A**). LDA and effect-size measurements identified the most differentially abundant taxons of the two groups (**B**). Comparison of the relative abundance at the bacterial family (**C**) and genus (**D**) levels in the samples from the two groups; * indicates p < 0.05; ^#^ indicates p < 0.01. Rotated principle-component analysis loading plot showing that the genera enriched in the LC patient samples could clustered together (**E**). Receiver operating-characteristic (ROC) curves for *Actinomyces* (**F**) and *Propionibacterium* (**G**) that were used to predict the presence of LC.

Figure 4A,B show the differences in the taxa represented in the CHB and LC oral bacterial communities and indicate the key phylotypes that could be used as microbiological markers at different phylogenetic levels to discriminate between CHB and LC patients. In contrast to the large differences in the compositions of the oral microbiota of healthy controls and patients with CHB or LC that were mentioned above, only a few microbial signatures of the oral microbiota of the CHB and LC groups differed. Specifically, at the phylum level, only Spirochaetes members were found in a significantly higher proportion in the CHB-patient samples. At the family level, members of *Neisseriaceae*, *Peptostreptococcaceae*, *Actinomycetaceae*, *Xanthomonadaceae* and *Nocardioidaceae* were significantly more abundant in the oral microbiota of the LC patients (Fig. 4C). At the genus level, the abundance of members of only several non-dominant genera, such as *Actinomyces*, *Filifactor*, *Solobacterium*, *Anaerovorax*, *Propionibacterium*, *Stenotrophomonas*, *Chryseobacterium*, *Delftia*, *Escherichia/Shigella*, *Flavobacterium*, *Paracoccus* and *Slackia*, was found to be significantly different in the CHB and LC groups (Fig. 4D). With the exception of the members of *Solobacterium*, the members of the other genera that were enriched in the LC-patient samples mostly clustered together in the rotated principle component analysis loading plot, which suggested that these genera could be used as potential microbiological biomarkers to discriminate LC patients from CHB patients (Fig. 4E). Receiver operating-characteristic (ROC) curves were generated for the different microbiota samples and the respective areas under the curve (AUC) were calculated (Fig. 4F,G). The values for the non-dominant genera, such as *Actinomyces*, *Propionibacterium*, *Stenotrophomonas* and *Chryseobacterium*, in the studied oral microbiota samples that reached an ideal cutoff point could distinguish the patients with LC from those with CHB. However, the usefulness of the oral microbiota values for these taxa in the differential diagnosis of LC was very limited because members of the non-abundant taxa were present in much lower proportions in the oral microbiota.

## Discussion

Similar to other bacterial habitats of the human body, such as the intestine, skin and vagina, the oral cavity harbours numerous indigenous microorganisms in a complex community that normally forms an equilibrium; however, pathogenesis can occur when the ecological balance is compromised. Our previous study demonstrated that the microbiota of a healthy human oral cavity is distinct from that of other bacterial habitats of the human body^17^. However, the microbiota in different sites of the oral cavity is far from uniform and its bacterial diversity level and composition varied dramatically among different sampling sites, such as the saliva, buccal mucosa, tongue dorsum, supragingival plaque and subgingival plaque^7^,^18^. Compared with the continuously varying salivary microbiota, the microbiota of the dental plaque, which consists of a dynamic and complex biofilm, represents an oral microbiota that is relatively stable over time, which has profound implications for the aetiology and progression of the most prevalent infections affecting humans, including dental caries^11^,^19^. A chronic HBV infection runs a long natural course during which underlying changes in liver tissue can result in the progression to cirrhosis and hepatic decompensation as well as to HCC^20^. The composition of the dental plaque of chronic HBV-infected patients can reflect the changing composition of the oral microbiota according to the course of the infection, which is why the supragingival plaque was selected for the analysis of the oral microbiota in our present study.

Using high-throughput pyrosequencing analysis, our present study provided knowledge of the oral microbiota of HBV-CLD patients at a much deeper level than previously achieved. With an average of 14,358 reads per sample and a nearly 99.0% Good’s coverage, a large number of members of rare taxa that were present in relatively low abundance were detected, which affected the observed overall bacterial diversity level of the tested oral microbiota. Compared with the overall bacterial diversity of the healthy-control samples, a decreased level was found in the samples obtained from HBV-CLD patients, which indicated that a chronic HBV infection could disturb the homeostasis of the oral cavity microbiota. In fact, a decreased bacterial diversity of the oral microbiota was found to impair the colonization resistance against invading pathogens, which eventually led to the dysbiosis of the oral cavity^21^. As the results of our previous study suggested, the dysbiosis of the oral microbiota might also contribute to alterations in the composition of the gut microbiota in patients with liver cirrhosis because autochthonous oral bacteria could easily break out of their oral microhabitats and freely invade the gut^2^. Bajaj *et al*. confirmed that the dysbiosis of the salivary microbiota reflected changes in the composition of the gut microbiota in patients with liver cirrhosis and hepatic encephalopathy^22^. In addition to the tested oral microbiota of patients with HBV-related cirrhosis, the corresponding samples obtained from patients with a chronic HBV infection were also included in present study, which allowed the analysis of the continuous and detailed change in the microbiota profiles according to the natural course of HBV infection. However, the alterations in the composition of the oral microbiota of LC patients were highly similar to those observed in samples obtained from CHB patients, which might be associated with the health status of these participants. The patients with CHB or LC could not be clearly separated according to the composition of their tested oral microbiota as determined using PCoA plotting. All of the LC patients enrolled in the present study were experiencing the compensated stage of the disease and did not exhibit hepatic encephalopathy, ascites or other complications. In fact, although HBV-related cirrhosis was the long-term outcome of a chronic HBV infection, the chronic and long-term course of an HBV infection might have contributed to the moderate alterations of the overall structure of the oral microbiota of chronic HBV-infected patients. Consistent with the results of a previous study of gut microbiota, the complications that accompanied other systemic diseases of the decompensated LC patients could dramatically alter the gut-liver axis microbiome and finally lead to changes in the composition of the oral microbiota^2^,^23^.

Generally, the level of oral malodour experienced in the breath of liver-disease patients is increased, which is mostly due to the presence of hydrogen sulphide (H_2_S)-, methyl mercaptan (CH_3_SH)- and dimethyl sulphide [(CH_3_)_2_S]-producing bacteria^24^,^25^. The results of a previous study demonstrated that distinct bacterial populations in the oral microbiota were involved in the production of high levels of H_2_S and CH_3_SH within the oral cavity^26^. Consistent with the observed differences in the gut microbiota compared with that of healthy subjects^2^,^3^, our present study demonstrated that the composition of the oral microbiota of patients with HBV-CLD was also significantly different from that of healthy subjects. In contrast to the composition of the salivary microbiota of oral-malodourous groups, an inversion of the Firmicutes/Bacteroidetes ratio, resulting in a higher proportion of Firmicutes organisms than of Bacteroidetes organisms, has been observed in patients with HBV-CLD^26^. The Firmicutes/Bacteroidetes ratio is considered to represent the health status of the human host and may reflect the eubiosis or dysbiosis of the oral cavity microbiota. Despite great inter-personal variations, the shifts in the Firmicutes/Bacteroidetes ratio were found to represent the level of dysbiosis of the oral microbiota after HBV infection, which might be associated with the translocation of bacteria from the oral cavity to the gut. Although the trend of the Firmicutes/Bacteroidetes ratio observed in the tested oral microbiota samples obtained from the LC patients was slightly lower than that of the CHB patients, these differences failed to reach the level of significance (P > 0.05). Consistent with the results of previous investigations, the results of our present study demonstrated that a higher proportion of potential H_2_S- and CH_3_SH-producers, such as members of *Porphyromonas*, *Treponema*, *Eubacterium*, *Solobacterium* and *Filifactor*, were observed in the CHB-group samples, whereas members of *Fusobacterium*, *Eubacterium*, *Parvimonas*, *Pseudomonas* and *Filifactor* were prevalent in the LC-group samples^22^,^26^,^27^. Members of most of the bacterial genera mentioned above were frequently detected in periodontal-disease samples and in dental caries, which implied that patients with CHB or LC were at a higher risk of the development of oral diseases. In contrast to the results of a previous study, the members of the genera that were abundant in the oral microbiota, such as those of *Leptotrichia* and *Prevotella*, which were reported to be positively correlated with the severity of oral malodour, were found to be dramatically decreased in patients with HBV-CLD^27^. In addition, the differentially altered patterns of the content of the potential H_2_S- and CH_3_SH-producers in the oral microbiota of the two HBV-CLD groups might be associated with the diverse array of malodorous compounds released, including that of various volatile sulphur-containing compounds, short-chain organic acids, diamines and phenyl compounds. These oral-malodourous associated bacteria could be potentially used as targets to evaluate and reduce the level of unpleasantness of the breath using antimicrobial compounds^28^.

Patients with a chronic HBV infection such as those with CHB and HBV-related LC are characterized by a weak immune response to HBV and an impaired immune response against this virus leads to chronic infection^29^. The immune response of these patients was impaired not only in their liver but also in other sites of their bodies, such as the oral cavity. The results of a previous study confirmed that patients with cirrhosis have an impaired oral defence system and a high level of inflammation, with significantly higher than normal levels of interleukin (IL)-6/IL-1β, and secretory IgA and lower than normal levels of lysozyme and histatins 1 and 5^22^. Wang *et al*. also demonstrated that the composition of the supragingival microbiota was more affected by the immune status of the host than by the gingivitis/periodontitis status^30^. The compromised immune response of patients with a chronic HBV infection might contribute to the dysbiosis of their oral microbiota^18^,^31^. Due to the dysbiosis of the oral microbiota of these patients, the oral microbiota-derived bacterial cells, such as those of *Fusobacterium* and *Treponema*, could easily break through the impaired oral defence system and invade the gut as opportunistic pathogens. These bacteria were also found in relatively higher proportions in the gut microbiota^2^,^32^,^33^. *Fusobacterium* is an oral bacterial group represented in the human microbiome, the members of which can invade tumour tissues and have been independently associated with a worse prognosis of pancreatic cancer^32^. *Treponema* is an oral anaerobic spirochete that is implicated in periodontal diseases and is also detected in gut microbiota^33^. Due to the relatively higher than normal abundance of members of *Fusobacterium* and *Treponema* in the HBV-CLD oral-microbiota samples, the levels of members of *Fusobacterium* and *Treponema* in the oral microbiota could be used for the differential diagnosis of HBV-CLD patients vs healthy controls (AUC = 0.770 and AUC = 0.860, respectively). Our present study provided additional data that confirmed our previously stated hypothesis that dysbiosis of the oral microbiota contributed to alterations in the composition of the gut microbiota of LC patients.

Several limitations in the present study should be acknowledged. First, our present descriptive study characterized only the oral microbiota of a particular site of patients with an HBV infection (as well as those of LC patients and heathy controls), whereas the gut microbiota of these participants were not simultaneously investigated and therefore, the data obtained was not appropriate to establish a direct linkage between the compositions of the oral and gut microbiota of these patients. The present study only provided the possibility that those oral-derived key phylotypes in HBV-CLD patients might contribute to the changes of gut microbiota. Second, the immune status of the HBV-CLD patients, particularly those of the gut mucosa and the oral cavity should be investigated in a future study, which would provide new correlations between the microbiota compositions and the immune responses of the human body. Such correlational studies might reveal that the modulation of the human microbiota actively re-established the functionality of the liver immune system, which facilitates viral clearance. Third, although the differences in the composition of the gut microbiota of HBV-CLD patients relative to those of other patients and healthy controls were investigated in our previous study^3^, future studies that characterize the oral microbiota of non-HBV-CLD patients will help to clarify the effects of an HB virus infection. Fourth, the sample size of the present study was relative small; more patients at each stage of the disease will make our conclusions more valid.

In summary, our present study demonstrated the deceased oral bacterial diversity in HBV-CLD patients. The change in the Firmicutes/Bacteroidetes ratio demonstrated that dysbiosis of the oral microbiota had occurred in both the CHB and the LC patients, although there was no significant different between the two groups. As our previous study suggested, alterations in the oral microbiota of HBV-CLD patients may contribute to the breakdown of their oral defences, resulting in key oral-derived phylotypes invading the gut. These key altered phylotypes could be used as non-invasive biomarkers for distinguishing HBV-CLD patients from healthy controls. In accompanying compromised immune responses, these changes in the oral microbiota revealed the dysbiosis involved in the development of HBV-CLD. Further investigations focused on the relationship between the restoration of the oral microbiota and HBV infections would help to clarify the role of the key bacterial phylotypes and provide new insight into the treatment of HBV-CLD.

## Methods

### Recruitment of the subjects

The protocols utilized in the present study were approved by the Ethics Committee of the First Affiliated Hospital, School of Medicine, Zhejiang University (China) and were implemented in accordance with the approved guidelines. Informed written consent was obtained from each of the participants prior to enrolment. Table 2 showed that thirty subjects were enrolled in this study, including 10 CHB patients, 10 patients with HBV-associated compensated liver cirrhosis (LC), and 10 healthy controls (HC). HBV-CLD participants included both 10 CHB patients and 10 HBV-LC patients. The diagnoses and disease grouping complied with “the guidelines for the prevention and treatment of chronic hepatitis B (2010 version)”^34^. Healthy control subjects were recruited from the people who visited the First Affiliated Hospital, School of Medicine, Zhejiang University for a routine physical examination. The following exclusion criteria were established: clinically apparent oral diseases, such as dental caries and periodontal disease; cigarette smoking; alcohol drinking; the use of antibiotics, probiotics, prebiotics, or synbiotics during the previous month; and known active bacterial, fungal, or other viral infections.

### Sample collection and extraction of bacterial genomic DNA

Sampling was performed in the morning before the participants had eaten breakfast. Skilled dentists collected the supragingival plaque samples by scraping the dental surfaces of the carious teeth approximately 20 times using a sterile metal loop. The metal loop was clipped off and immersed in 1 ml of a saline solution that had been UV-irradiated to prevent DNA contamination. Theses samples were immediately placed in liquid nitrogen and were stored at −80 °C until use. The plaque samples were suspended and then were pelleted by centrifugation (Thermo Electron Corporation, Boston, MA, USA) at full speed (>10,000 × g) for 10 min. The bacterial genomic DNA was extracted using a QIAamp DNA Mini Kit (QIAGEN, Hilden, Germany) as described previously, with modifications^11^,^19^. All of the DNA was stored at −20 °C before further analysis.

### Barcoded pyrosequencing analyses

The bacterial genomic DNA was utilized in 50-μl triplicate samples for the specific amplification of the V1-V3 hypervariable regions of the 16S rRNA gene using the 27F (5′-AGAGTTTGATCCTGGCTCAG-3′) and 533R (5′-TTACCGCGGCTGCTGGCAC-3′) primers, as described in our previous reports^35^,^36^. Each forward primer incorporated FLX Titanium adapters and a sample barcode was incorporated at the 5′ end of the reverse primer to allow all of the samples to be included in the single 454 FLX sequencing run. After the PCR products were extracted and quantified, they were pooled in equimolar concentrations and were sequenced using a 454 Life Sciences Genome Sequencer FLX system (Roche, Basel, Switzerland) according to the manufacturer’s recommendations. The methods for sequence screening, diversity analysis and taxonomy-based analysis were performed as described in our previous reports^35^,^36^. The Mann–Whitney *U* test and a one-way ANOVA were used for statistical analysis, and all of these analyses were performed using SPSS for Windows software (version 16.0; SPSS Inc., Chicago, IL, USA).

## Additional Information

**Accession numbers**: The sequence data from this study has been deposited in the GenBank Sequence Read Archive with the accession number SRP058372.

**How to cite this article**: Ling, Z. *et al*. Decreased Diversity of the Oral Microbiota of Patients with Hepatitis B Virus-Induced Chronic Liver Disease: A Pilot Project. *Sci. Rep*. **5**, 17098; doi: 10.1038/srep17098 (2015).

## Supplementary Material

Supplementary Information

## Figures and Tables

**Table 1:** Comparison of phylotype coverage and diversity estimation of the 16S rRNA gene libraries at 97% similarity from the pyrosequencing analysis.

Group	No. of Reads	No. of OTUs^1^	Good’s^2^	Richness estimator				Diversity index	
				ACE	95% CI	Chao1	95% CI	Shannon	Simpson
HC	118480	4599	97.78%	17854	16961.3–18746.7	11029	10477.6–11580.5	5.938522	0.007557
CHB	150023	3976	98.68%	11678	11094.1–12261.9	7910	7514.5–8305.5	5.635272	0.013999
LC	162250	4096	98.79%	10722	10185.9–11258.1	7661	7278.0–8044.1	5.469637	0.015747

^1^The operational taxonomic units (OTUs) were defined at the 97% similarity level.

^2^The coverage percentage (Good’s), richness estimators (ACE and Chao1) and diversity indices (Shannon and Simpson) were calculated using Good’s method and the mothur program, respectively.

**Table 2:** Descriptive data of healthy control subjects and patients in the study.

	Healthy Control (n = 10)	Chronic hepatitis B patients (n = 10)	HBV-associated cirrhotic patients (n = 10)
Age (years)	48 ± 11	43 ± 10	49 ± 9
Gender (M/F)	6/4	5/5	6/4
BMI (kg/m^2^)	23.5 ± 3.4	22.8 ± 3.2	22.2 ± 2.9
ALB (g/L)	43.3 ± 4.3	42.8 ± 3.9	39.4 ± 6.9^*^
ALT (U/L)	23.8 ± 11.2	259.0 ± 208.9^*^	49.6 ± 44.8^*,#^
AST (U/L)	25.7 ± 8.4	183.4 ± 125.5^*^	68.7 ± 38.2^*,#^
TBIL (μmol/L)	11.3 ± 3.4	63.5 ± 32.5^*^	59.8 ± 45.7^*^
CHE (U/L)	7332 ± 3450	7015 ± 2895	5839 ± 3893^*,#^
PT (s)	12.7 ± 4.2	13.2 ± 4.1	14.3 ± 5.3
HBeAg+(no.)	0	10	10
HBV-DNA (log_10_ copies/mL, range)	0	4.5 (3.8–5.8)	4.0 (3.5–5.5)

Abbreviations: BMI: body mass index; ALB: serum albumin; ALT: alanine transarninase; AST: aspartate aminotransferase; CHE: cholinesterase; HBV: hepatitis B virus; PT: prothrombin time; TBIL: total bilirubin. no.: numbers. *P* values are based on chi squared and ANOVA test where appropriate. *P* < 0.05 was labeled; ^*^compared with healthy control; ^#^compared with chronic hepatitis B patients.

## References

[b1] Bajaj J. S. . Altered profile of human gut microbiome is associated with cirrhosis and its complications. J Hepatol 60, 940–947 (2014). 24374295 10.1016/j.jhep.2013.12.019 PMC3995845

[b2] Qin N. . Alterations of the human gut microbiome in liver cirrhosis. Nature 513, 59–64 (2014). 25079328 10.1038/nature13568

[b3] Chen Y. . Characterization of fecal microbial communities in patients with liver cirrhosis. Hepatology 54, 562–572 (2011). 21574172 10.1002/hep.24423

[b4] Wei X. . Abnormal fecal microbiota community and functions in patients with hepatitis B liver cirrhosis as revealed by a metagenomic approach. BMC Gastroenterol 13, 175 (2013). 24369878 10.1186/1471-230X-13-175 PMC3878425

[b5] Schnabl B. & Brenner D. A. Interactions Between the Intestinal Microbiome and Liver Diseases. Gastroenterology 146, 1513–1524 (2014). 24440671 10.1053/j.gastro.2014.01.020 PMC3996054

[b6] Chou H. H. . Age-related immune clearance of hepatitis B virus infection requires the establishment of gut microbiota. Proc Natl Acad Sci USA 112, 2175–2180 (2015). 25646429 10.1073/pnas.1424775112 PMC4343154

[b7] Aas J. A., Paster B. J., Stokes L. N., Olsen I. & Dewhirst F. E. Defining the normal bacterial flora of the oral cavity. J Clin Microbiol 43, 5721–5732 (2005). 16272510 10.1128/JCM.43.11.5721-5732.2005 PMC1287824

[b8] Crielaard W. . Exploring the oral microbiota of children at various developmental stages of their dentition in the relation to their oral health. BMC Med Genomics 4, 22 (2011). 21371338 10.1186/1755-8794-4-22 PMC3058002

[b9] Curtis M. A., Zenobia C. & Darveau R. P. The relationship of the oral microbiotia to periodontal health and disease. Cell Host Microbe 10, 302–306 (2011). 22018230 10.1016/j.chom.2011.09.008 PMC3216488

[b10] Schmidt B. L. . Changes in Abundance of Oral Microbiota Associated with Oral Cancer. PLoS One 9, e98741 (2014). 24887397 10.1371/journal.pone.0098741 PMC4041887

[b11] Ling Z. X. . Analysis of Oral Microbiota in Children with Dental Caries by PCR-DGGE and Barcoded Pyrosequencing. Microb Ecol 60, 677–690 (2010). 20614117 10.1007/s00248-010-9712-8

[b12] Said H. S. . Dysbiosis of Salivary Microbiota in Inflammatory Bowel Disease and Its Association With Oral Immunological Biomarkers. DNA Research 21, 15–25 (2014). 24013298 10.1093/dnares/dst037 PMC3925391

[b13] Farrell J. J. . Variations of oral microbiota are associated with pancreatic diseases including pancreatic cancer. Gut 61, 582–588 (2012). 21994333 10.1136/gutjnl-2011-300784 PMC3705763

[b14] Zeigler C. C. . Microbiota in the Oral Subgingival Biofilm Is Associated With Obesity in Adolescence. Obesity 20, 157–164 (2012). 21996660 10.1038/oby.2011.305

[b15] Koren O. . Human oral, gut, and plaque microbiota in patients with atherosclerosis. Proc Natl Acad Sci USA 108, 4592–4598 (2011). 20937873 10.1073/pnas.1011383107 PMC3063583

[b16] Sheehy E. C., Beighton D. & Roberts G. J. The oral microbiota of children undergoing liver transplantation. Oral Microbiol Immun 15, 203–210 (2000). 10.1034/j.1399-302x.2000.150309.x 11154404

[b17] Ling Z. . Pyrosequencing analysis of the human microbiota of healthy Chinese undergraduates. BMC Genomics 14, 390 (2013). 23758874 10.1186/1471-2164-14-390 PMC3685588

[b18] Lin C. Y. . Endotoxemia contributes to the immune paralysis in patients with cirrhosis. J Hepatol 46, 816–26 (2007). 17328986 10.1016/j.jhep.2006.12.018

[b19] Jiang W. . Pyrosequencing analysis of oral microbiota shifting in various caries states in childhood. Microb Ecol 67, 962–9 (2014). 24504329 10.1007/s00248-014-0372-y

[b20] Hadziyannis S. J., Vassilopoulos D. & Hadziyannis E. The natural course of chronic hepatitis B virus infection and its management. Adv Pharmacol 67, 247–91 (2013). 23886003 10.1016/B978-0-12-405880-4.00007-X

[b21] Ling Z. . Molecular analysis of the diversity of vaginal microbiota associated with bacterial vaginosis. BMC Genomics 11, 488 (2010). 20819230 10.1186/1471-2164-11-488 PMC2996984

[b22] Basic A. & Dahlen G. Hydrogen sulfide production from subgingival plaque samples. Anaerobe 35, 21–27 (2015). 25280920 10.1016/j.anaerobe.2014.09.017

[b23] Chassaing B., Etienne-Mesmin L. & Gewirtz A. T. Microbiota-liver axis in hepatic disease. Hepatology 59, 328–339 (2014). 23703735 10.1002/hep.26494 PMC4084781

[b24] Han D. H., Lee S. M., Lee J. G., Kim Y. J. & Kim J. B. Association between viral hepatitis B infection and halitosis. Acta Odontol Scand 72, 274–282 (2014). 24053367 10.3109/00016357.2013.823645

[b25] Van den Velde S., Nevens F., Van Hee P., van Steenberghe D. & Quirynen M. GC-MS analysis of breath odor compounds in liver patients. J Chromatogr B Analyt Technol Biomed Life Sci 875, 344–348 (2008). 10.1016/j.jchromb.2008.08.031 18938115

[b26] Takeshita T. . Discrimination of the oral microbiota associated with high hydrogen sulfide and methyl mercaptan production. Sci Rep 2, 215 (2012). 22355729 10.1038/srep00215 PMC3253589

[b27] Yang F. . Microbial basis of oral malodor development in humans. J Dent Res 92, 1106–1112 (2013). 24101743 10.1177/0022034513507065

[b28] Dadamio J. . Efficacy of different mouthrinse formulations in reducing oral malodour: a randomized clinical trial. J Clin Periodontol 40, 505–513 (2013). 23489103 10.1111/jcpe.12090

[b29] Stoop J. N. . Regulatory T cells contribute to the impaired immune response in patients with chronic hepatitis B virus infection. Hepatology 41, 771–778 (2005). 15791617 10.1002/hep.20649

[b30] Wang Y. . Oral microbiota distinguishes acute lymphoblastic leukemia pediatric hosts from healthy populations. PLoS One 9, e102116 (2014). 25025462 10.1371/journal.pone.0102116 PMC4099009

[b31] Albillos A., Lario M. & Alvarez-Mon M. Cirrhosis-associated immune dysfunction: distinctive features and clinical relevance. J Hepatol 61, 1385–1396 (2014). 25135860 10.1016/j.jhep.2014.08.010

[b32] Mitsuhashi K. . Association of *Fusobacterium* species in pancreatic cancer tissues with molecular features and prognosis. Oncotarget 6, 7209–7220 (2015). 25797243 10.18632/oncotarget.3109 PMC4466679

[b33] De Filippo C. . Impact of diet in shaping gut microbiota revealed by a comparative study in children from Europe and rural Africa. Proc Natl Acad Sci USA 107, 14691–14696 (2010). 20679230 10.1073/pnas.1005963107 PMC2930426

[b34] Chinese Society of Hepatology and Chinese Society of Infectious Diseases, C.M.A. The guideline of prevention and treatment for chronic hepatitis B (2010 version). Zhonghua Gan Zang Bing Za Zhi 19, 13–24 (2011). (in Chinese). 21272453 10.3760/cma.j.issn.1007-3418.2011.01.007

[b35] Ling Z. . Altered fecal microbiota composition associated with food allergy in infants. Appl Environ Microbiol 80, 2546–54 (2014). 24532064 10.1128/AEM.00003-14 PMC3993190

[b36] Ling Z. . Impacts of infection with different toxigenic Clostridium difficile strains on faecal microbiota in children. Sci Rep 4, 7485 (2014). 25501371 10.1038/srep07485 PMC4265774

