# Supplementary material for: Decreased Diversity of the Oral Microbiota of Patients with Hepatitis B Virus-Induced Chronic Liver Disease: A Pilot Project
Source: Sci Rep. 2015 Nov 26;5:17098. doi: 10.1038/srep17098 (PMC4660595; doi:10.1038/srep17098)
Supplement: Supplementary Information [file srep17098-s1.pdf]

Supplementary information

Decreased Diversity of the Oral Microbiota of Patients with Hepatitis B Virus-Induced Chronic Liver Disease

Zongxin Ling[1]*, Xia Liu[1,2]*, Yiwen Cheng[1], Xiawei Jiang[1], Haiyin Jiang[1], Yuezhu Wang[3], Lanjuan Li[1#]

[1] Collaborative Innovation Center for Diagnosis and Treatment of Infectious Diseases, State Key Laboratory for Diagnosis and Treatment of Infectious Diseases, the First Affiliated Hospital, School of Medicine, Zhejiang University, Hangzhou, Zhejiang, 310003, China;

[2] Intensive Care Unit, the First Affiliated Hospital, School of Medicine, Zhejiang University, Hangzhou, Zhejiang, 310003, China;

[3] Shanghai-MOST Key Laboratory of Health and Disease Genomics, Chinese National Human Genome Center at Shanghai, Shanghai 201203, China;

*These authors contribute equally to this work.

Running title: Decreased oral bacterial diversity in HBV-CLD

[#]**Corresponding author**:

Prof. Lanjuan Li, State Key Laboratory for Diagnosis and Treatment of Infectious Diseases, the First Affiliated Hospital, School of Medicine, Zhejiang University, Hangzhou, Zhejiang, 310003, China. E-mail: ljli@zju.edu.cn. Tel.: +86-571-8723-6458; Fax: +86-571-8723- 6459.

Figure S1 Rarefaction analysis of the pyrosequencing tags identified in the oral microbiota derived from CHB (n=10) and LC (n=10) patients and healthy controls (n=10).

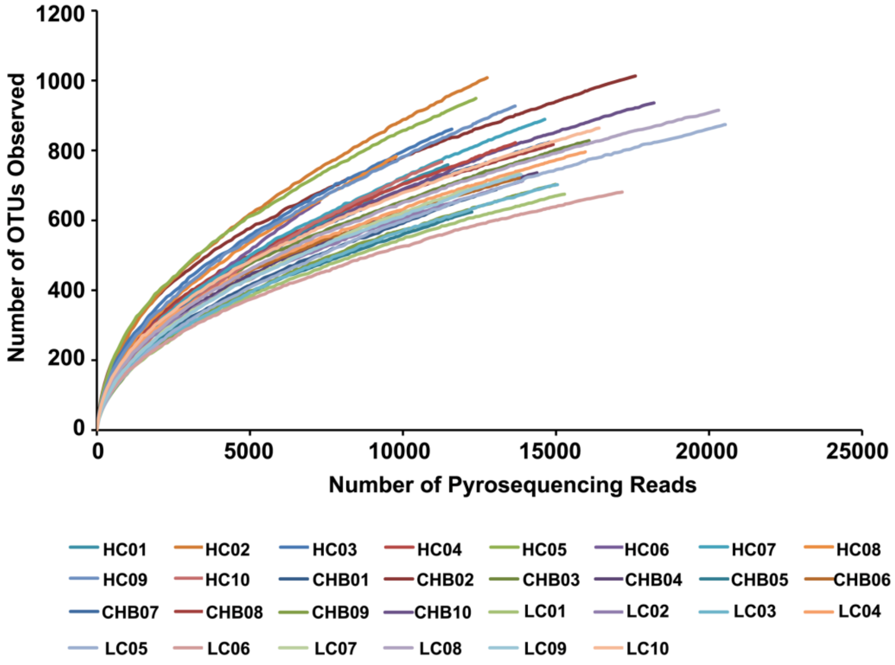

Figure S2 LEfSe was used to identify the most differentially abundant taxa of the healthy control and HBV-CLD patient samples. (Red) HBV-CLD-enriched taxa; (Green) healthy control-enriched taxa. The brightness of each dot is proportional to its effect size (A). The healthy control-enriched taxa are indicated with a positive LDA score (green) and the HBV-CLD-enriched taxa have a negative score (red). Only the taxa meeting a significant LDA threshold value of >2 are shown (B).

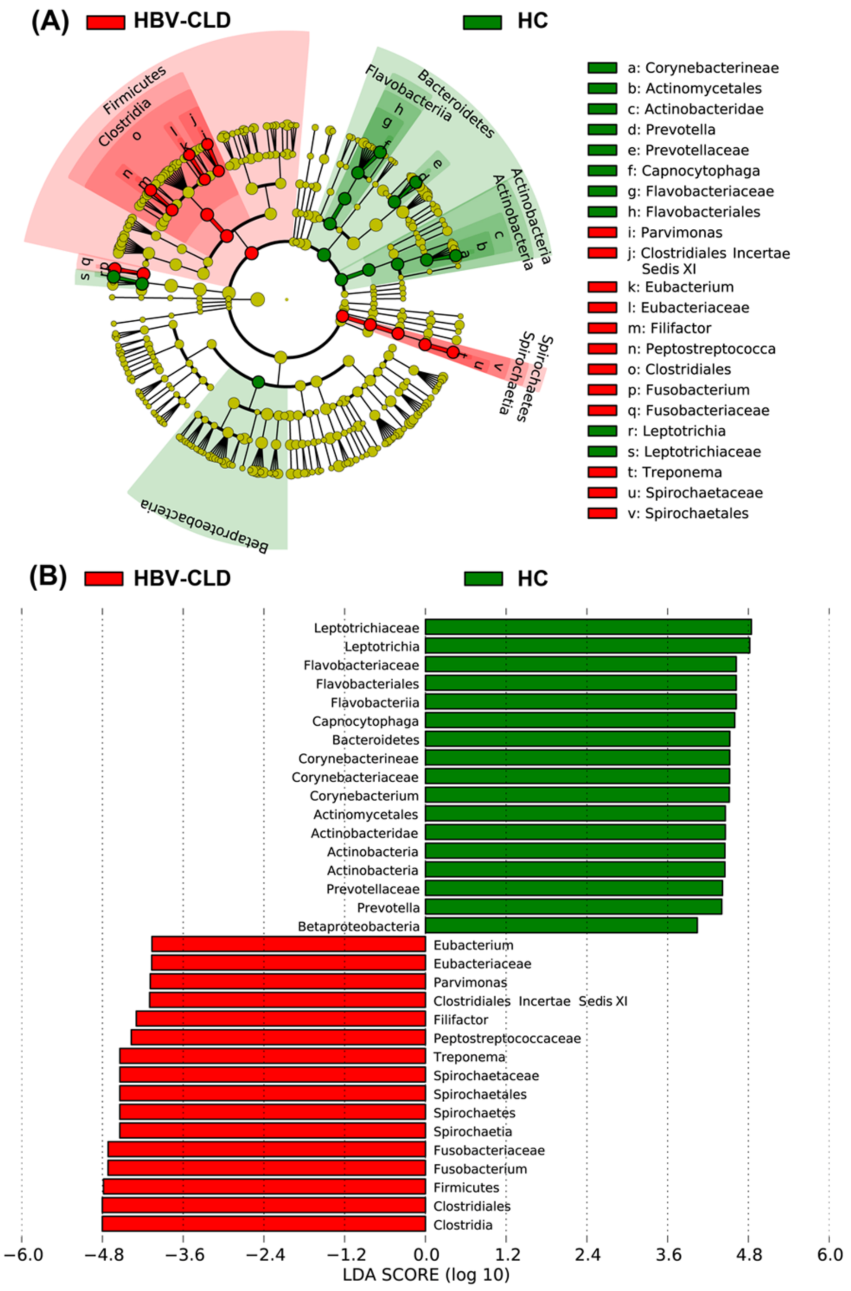

Figure S3 LEfSe was used to identify the most differentially abundant taxa of the healthy control and LC patient samples. (Green) LC-enriched taxa; (Red) healthy control-enriched taxa. The brightness of each dot is proportional to its effect size (A). The healthy control-enriched taxa are indicated with a positive LDA score (red), and taxa enriched in CHB have a negative score (green). Only the taxa meeting a significant LDA threshold value of >2 are shown (B). Comparison of the relative abundance at the bacterial phylum (C), order (D), family (E) and genus (F) levels in the HC and LC groups; * indicates p < 0.05; # indicates p < 0.01.

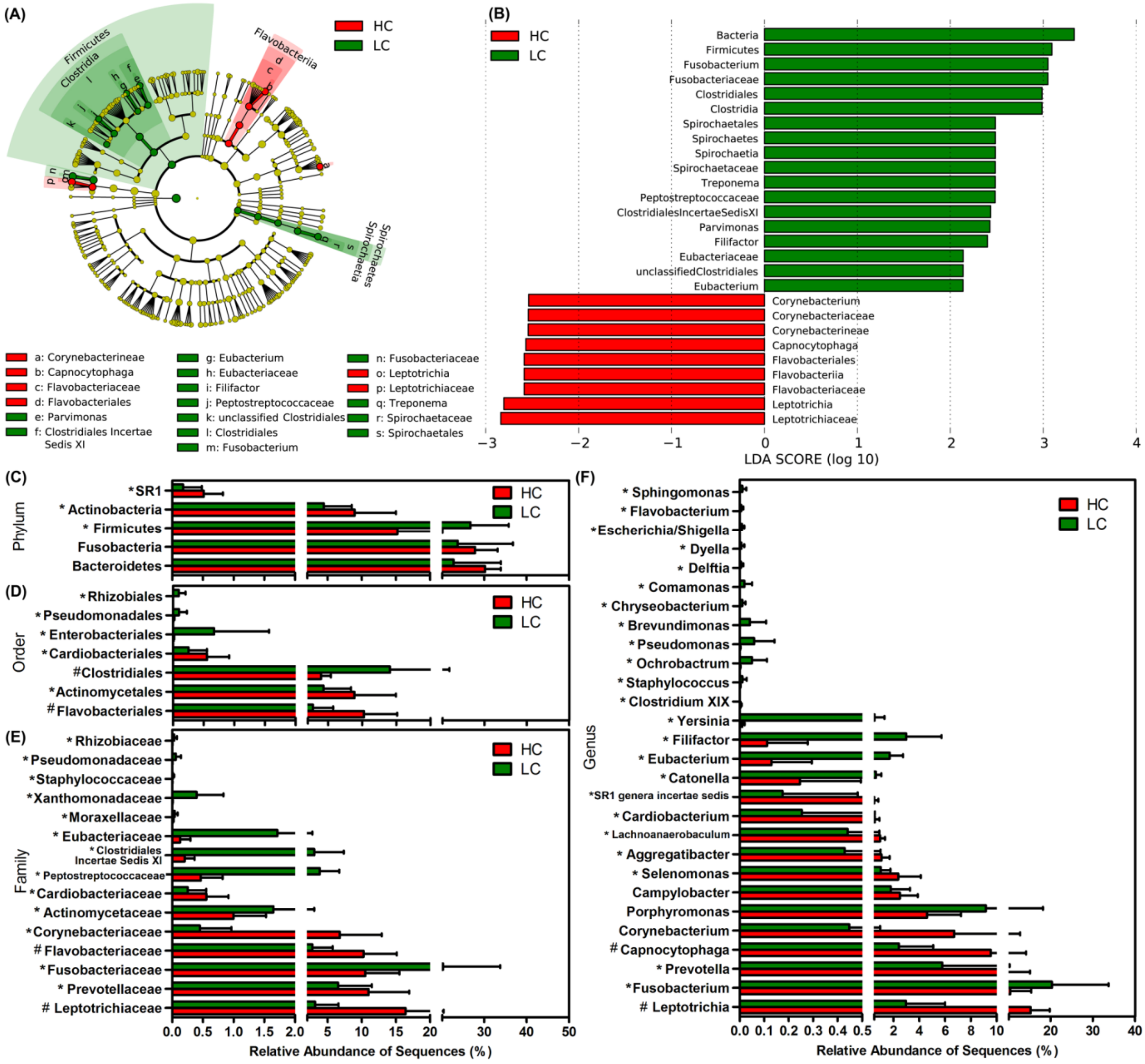

Table S1 Comparison of the phylotype coverages and the diversity estimates of the 16S rRNA gene libraries of individuals at a 97% similarity level, as determined using pyrosequencing analysis

Sample ID	No. of Reads	No. of OTUs	Good's	Richness estimator		Diversity index	
				ACE	Chao 1	Shannon	Simpson
HC01	11467	759	96.38%	2637.0	1601.2	4.834598	0.016921
HC02	12750	1008	95.78%	3564.3	2201.8	5.102169	0.016301
HC03	11596	861	96.03%	3096.9	1949.4	4.993331	0.018050
HC04	13678	822	96.87%	2602.4	1794.1	4.751028	0.022691
HC05	12381	949	96.15%	2973.6	1981.1	5.153942	0.016561
HC06	7266	652	94.30%	3194.6	1807.3	4.178176	0.047066
HC07	14633	889	96.63%	3389.4	2011.9	4.730115	0.022018
HC08	9779	783	95.47%	3070.8	1883.0	4.801959	0.021066
HC09	13663	927	96.35%	3089.2	2121.7	4.959427	0.016976
HC10	11267	768	96.05%	2761.6	1841.8	4.376678	0.032434
CHB01	13048	688	97.13%	2596.3	1570.9	4.333613	0.035285
CHB02	17592	1013	97.26%	2912.0	2127.6	5.348895	0.009905
CHB03	16080	828	97.48%	2438.2	1552.0	4.685375	0.024890
CHB04	14378	736	97.54%	1891.4	1376.5	4.335313	0.039698
CHB05	12250	624	97.34%	2063.2	1439.0	4.166275	0.056167
CHB06	13798	722	97.54%	1961.4	1388.2	4.466473	0.048711
CHB07	14785	824	97.17%	2478.3	1830.6	4.580713	0.038681
CHB08	14908	817	97.53%	1867.2	1460.1	4.215734	0.093006
CHB09	14984	703	97.57%	1897.5	1595.8	3.973302	0.051159
CHB10	18200	936	97.45%	2597.6	1820.3	4.715659	0.025353
LC01	15276	675	97.81%	1740.8	1218.2	3.948043	0.064233
LC02	13909	713	97.47%	2092.6	1457.3	4.454149	0.034325
LC03	15046	702	97.58%	2226.8	1343.4	4.447291	0.032099
LC04	15955	796	97.54%	2399.7	1629.0	4.530514	0.036119
LC05	20529	874	97.83%	2374.3	1577.8	3.954720	0.056942
LC06	17160	681	98.05%	1901.6	1355.0	3.852604	0.079112
LC07	13806	733	97.27%	2040.4	1495.1	4.353164	0.031768
LC08	20311	915	97.81%	2266.8	1699.0	4.121474	0.069421
LC09	13853	731	97.31%	1998.3	1542.8	4.185983	0.043478
LC10	16405	864	97.41%	2384.5	1675.7	4.588300	0.042965
